# Automated line-clearing chest tubes reduce postoperative pain and atrial fibrillation after cardiac surgery

**DOI:** 10.1016/j.xjon.2024.09.019

**Published:** 2024-09-24

**Authors:** Elbert E. Heng, Oluwatomisin Obafemi, Danielle Mullis, Alyssa Garrison, Hanjay Wang, Jack H. Boyd

**Affiliations:** Department of Cardiothoracic Surgery, Stanford University School of Medicine, Stanford, Calif

**Keywords:** atrial fibrillation, cardiac surgery, chest tube, enhanced recovery, perioperative, postoperative pain

## Abstract

**Objective:**

Recent advancements in chest tube technologies have gained interest for their ability to enhance postoperative recovery via reduction of retained blood syndrome after cardiothoracic surgery. The present study investigates the effect of the Centese Thoraguard automated line-clearance chest tube system on postoperative pain and recovery after cardiac surgery.

**Methods:**

This was a single-center retrospective review of 1771 adult patients undergoing nonemergency cardiac surgery between January 2021 and December 2022. Perioperative data were analyzed in 184 patients undergoing surgery with Thoraguard automated clearance chest tubes and 1587 patients with conventional chest tubes. Postoperative outcomes were compared in a propensity-matched cohort of 133 patient pairs with similar preoperative characteristics.

**Results:**

Patients undergoing cardiac surgery with automated clearance chest tubes demonstrated significant reductions in pain scores (0-10) compared with conventional chest tubes on the third postoperative day (5 vs 6, *P* = .02) and at hospital discharge (0 vs 3, *P* = .04). Automated clearance chest tubes were associated with a shorter time on the ventilator (5.3 vs 5.8 hours, *P* < .001). There was a significant reduction in postoperative atrial fibrillation (18.1% vs 30.8%, *P* = .02) in patients receiving automated clearance chest tubes. There were no significant differences in mortality, myocardial infarction, or stroke between automated line-clearing and conventional chest tubes.

**Conclusions:**

The use of the Thoraguard automated line-clearing chest tube system in routine cardiac surgery was associated with improved postoperative pain control, decreased ventilator duration, and decreased postoperative atrial fibrillation without increased morbidity or mortality.

**Figure:**
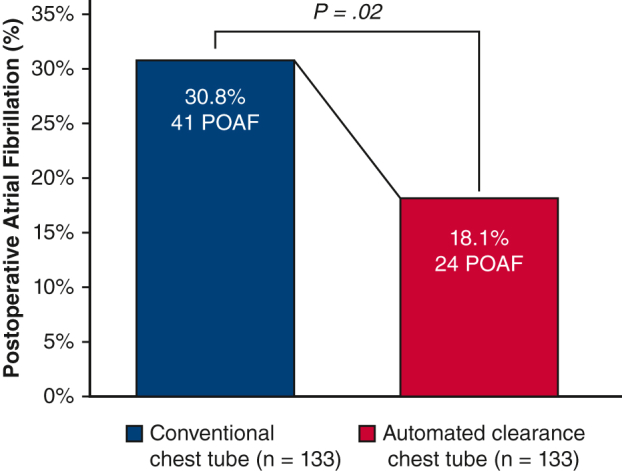
Automated line-clearing chest tubes reduce POAF.

**Central Message**

This study demonstrates that the Centese Thoraguard automated clearance chest tube system enhances patient recovery by reducing postoperative pain and atrial fibrillation after cardiac surgery.

**Perspective**

Active clearance chest tubes have the potential to mitigate RBS and enhance patient recovery after cardiac surgery. In this propensity-matched analysis, we share our institutional experience with the use of the Centese Thoraguard automated line-clearance chest tube system and demonstrate significant reductions in postoperative pain and atrial fibrillation after cardiac surgery.

Chest tube drainage is a well-established component of cardiothoracic surgery and is essential to the postoperative management and recovery of patients after surgery. Obstruction of mediastinal chest tubes is a common issue occurring in approximately 36% of cases and presents a clinical challenge in the form of retained blood syndrome (RBS).^1^^,^^2^ After chest tube obstruction by clotted blood, retained blood in the mediastinum serves as an inflammatory nidus associated with increased postoperative complications, including atrial fibrillation, prolonged mechanical ventilation, and mortality from cardiac tamponade.^3^^,^^4^ In an effort to minimize complications and facilitate postoperative recovery, the maintenance of chest tube patency has been given a class I recommendation in the Enhanced Recovery After Surgery Society Guidelines for Perioperative Care in Cardiac Surgery.^5^

In recent years, advancements in chest tube technologies have gained interest for their potential to enhance postoperative recovery via reduction of RBS. Although the efficacy of traditional methods, such as stripping or external suctioning, for clearing obstructed chest tubes has been called into question, active clearance chest tube systems have been designed as a potential solution to maintain tube patency without physical manipulation or compromise to the sterile field. At our institution, we previously published the first-in-human study of the Centese Thoraguard automated line-clearing chest tube system demonstrating its safety profile and drainage efficacy in routine cardiac surgery.^6^ In the present study, we examine our institutional experience to investigate the effect of this automated clearance chest tube system on postoperative pain and recovery compared with conventional chest tubes after cardiac surgery.

## Material and Methods

### Study Design

This was a single-center retrospective review of 1771 adult patients undergoing nonemergency cardiac surgery at our institution between January 1, 2021, and December 31, 2022. The study was approved by the Institutional Review Board at Stanford University (IRB68619 approved November 29, 2023), and a waiver of consent was granted. Patients who received an automated clearance chest drainage system were grouped and compared with patients who received conventional chest tubes after cardiac surgery. Exclusion criteria included patients aged less than 18 years and those undergoing emergency cardiac surgery. Medical records were reviewed for preoperative patient characteristics, intraoperative details, and postoperative outcomes during the hospital stay. Patient pain scores were recorded on the Integer Rating Scale from 0 to 10 and reported in the Society of Thoracic Surgeons (STS) Adult Cardiac Surgery Database version 4.20 for the highest recorded scores at baseline, on postoperative day 3, and on day of discharge. Postoperative atrial fibrillation (POAF) was defined as new-onset atrial fibrillation after surgery lasting more than 1 hour or atrial fibrillation lasting less than 1 hour but requiring medical or procedural intervention.

### Automated Chest Drainage System

**Figure 1:**
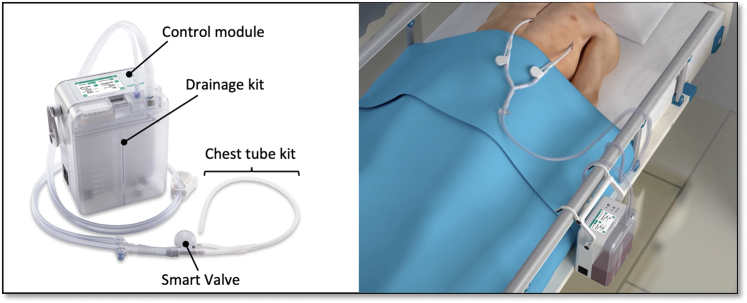
Thoraguard automated line-clearance chest tube system and components (*left*). Bedside device positioning and integrated suction without wall vacuum (*right*).

**Figure 2:**
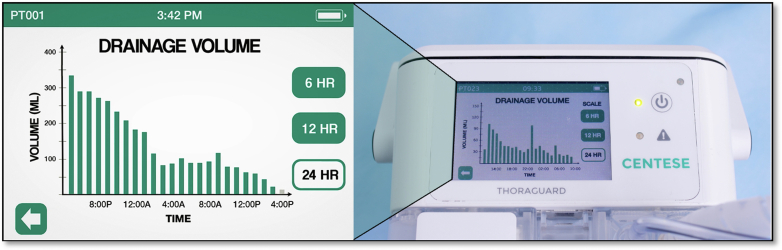
Thoraguard Control Module display with automated digital recording of hourly chest tube outputs over 24 hours.

The Thoraguard System is a digital and automated line-clearance chest drainage system developed by Centese and the Food and Drug Administration cleared through the 510K process. The system includes 3 components: the Thoraguard Drainage Kit, Thoraguard Control Module, and Thoraguard Chest Tube Kit (Figure 1). The control module comprises an electronic monitor with an integrated pump, battery, and sensors to regulate suction and digitally measure fluid output and air leaks (Figure 2). The drainage kit includes a 1200-mL canister and connection tubing, and the chest tube kit consists of a 20F dual-lumen drainage catheter and a SmartValve filter to allow for automated clearance. Automated line clearance is achieved by transiently increasing suction from −20 cm H_2_O to −100 cm H_2_O every 5 minutes, triggering the SmartValve to pull ambient air through a sterilization-grade filter and generate an air bolus that sweeps fluid from the chest tube into the drainage canister.

### Conventional Chest Tube System

The conventional chest drainage system used at our institution is the Atrium Oasis Dry Suction Water Seal Chest Drain manufactured by Getinge. Conventional chest tubes connected to the Atrium were supplied by Covidien and ranged from 24F to 40F in size.

### Statistical Analysis

**Figure E1:**
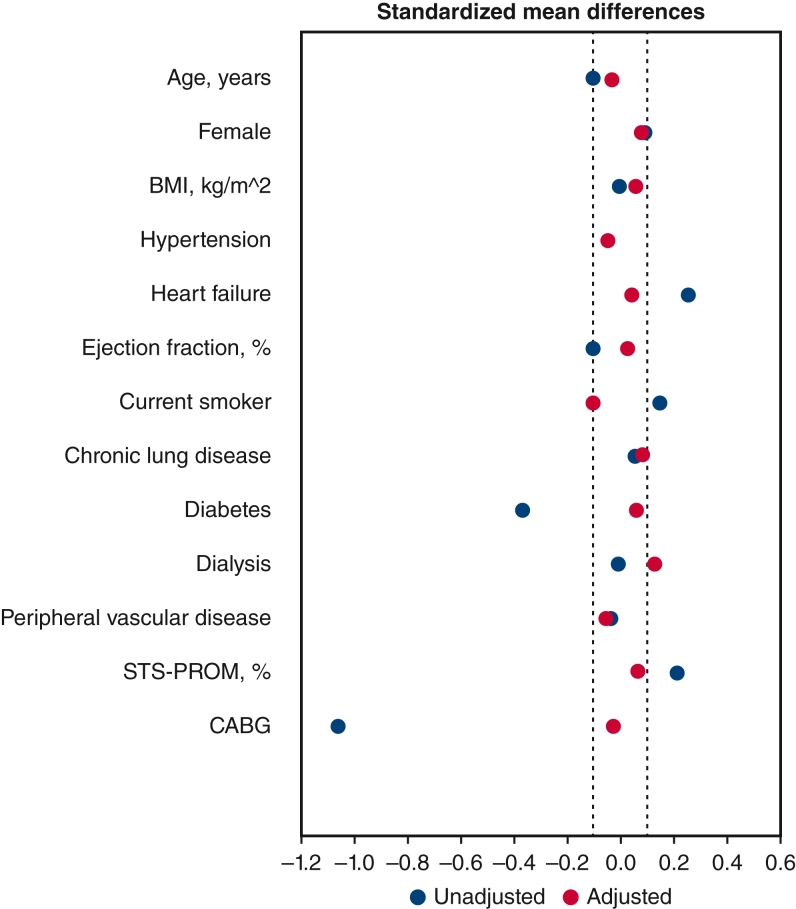
Covariate balance between groups before (unadjusted) and after (adjusted) propensity matching. *BMI*, Body mass index; *STS-PROM*, Society of Thoracic Surgeons Predicted Risk of Mortality; *CABG*, coronary artery bypass grafting.

Patients receiving automated clearance chest tubes (n = 184) were propensity matched 1:1 to patients receiving conventional chest tubes (n = 1587). The propensity score for receiving automated clearance chest tubes was calculated with logistic regression using the following patient variables: age, sex, body mass index, hypertension, heart failure, ejection fraction, current smoking status, chronic lung disease, diabetes, dialysis, peripheral vascular disease, STS Predicted Risk of Mortality, and planned coronary artery bypass grafting (CABG). Patients with similar propensity scores were matched 1:1 in a nearest neighbor fashion without replacement using a caliper width of 0.1 in the “psmatch2” statistical package in Stata.^7^ Propensity matching yielded a well-matched cohort of n = 133 patient pairs. Covariate balance was assessed by standardized mean differences between groups before and after matching (Figure E1). Data are presented as counts with percentages or median with interquartile range (IQR). Between group differences were assessed using Pearson’s chi-square or Fisher exact tests for categorical variables and 2-tailed Student *t* test or Mann–Whitney *U* test for continuous variables. All statistical analyses were performed using STATA/SE v17.0 (StataCorp LLC).

## Results

### Patient Characteristics

Over the 2-year study period, a total of 184 patients underwent cardiac surgery with placement of automated line-clearing chest tubes, and 1587 patients underwent cardiac surgery with placement of conventional chest tubes. The most common operation performed with automated clearance chest tubes was isolated CABG (n = 122/184, 66%) followed by myocardial bridge unroofing (n = 22/184, 12.0%) (Table E1). Patients receiving automated clearance chest tubes had a mean age of 63.4 ± 12.5 years, with 29.9% being female (Table E2). Compared with patients receiving conventional chest tubes, patients receiving automated clearance chest tubes had a lower incidence of heart failure (37.5% vs 50.2%; *P* < .01) and a higher incidence of diabetes (42.9% vs 25.6%; *P* < .001).

### Propensity-Matched Cohort

Propensity matching on preoperative characteristics yielded a matched study cohort of 133 patient pairs between the automated clearance chest tube and conventional chest tube groups. After matching, isolated CABG remained the most common operation performed at an equal rate between both groups (113/133 automated clearance, 85.0% and 113/133 conventional, 85.0%) (Table 1). Patients receiving automated clearance chest tubes and those receiving conventional chest tubes had similar baseline clinical characteristics including age, sex, body mass index, history of heart failure, and history of diabetes. Ejection fraction (55.7% ± 6.8% vs 56.0% ± 9.4%; *P* = .81) and STS Predicted Risk of Mortality (0.9%; IQR, 0.5-2.1 vs 0.9%; IQR, 0.5-1.7; *P* = .95) were similar between automated clearance and conventional chest tube groups (Table 2).

**Table 1:** Operative case breakdown (matched)

Operation	Conventional patients (n = 133)	Automated clearance (n = 133)	Total (n = 266)
CABG	113	113	226
AVR	0	1	1
MVR	3	4	7
AVR/CABG	1	5	6
MVR/CABG	2	1	3
MVr	6	4	10
MVr/CABG	3	2	5
MVR/TVr	1	2	3
MVr/TVr	3	1	4
MVR/TVr/CABG	1	0	1

*CABG*, Coronary artery bypass grafting; *AVR*, aortic valve replacement; *MVR*, mitral valve replacement; *MVr*, mitral valve repair; *TVr*, tricuspid valve repair.

**Table 2:** Baseline patient characteristics (matched)

Variable	Conventional patients (n = 133)	Automated clearance (n = 133)	*P* value
Age, y	65 ± 10.7	66 ± 9.9	.77
Female	39 (29.3%)	34 (25.6%)	.58
BMI, kg/m^2^	28.3 ± 5.4	28.0 ± 5.89	.64
Hypertension	119 (89.5)	121 (91.0)	.84
Heart failure	53 (39.9%)	50 (37.6%)	.80
Ejection fraction, %	56.0 ± 9.4	55.7 ± 10.9	.81
Current smoker	6 (4.5%)	9 (6.8%)	.60
Chronic lung disease	21 (15.8%)	17 (12.8%)	.60
Diabetes	72 (54.1%)	68 (51.3%)	.71
Dialysis	10 (7.5%)	6 (4.5%)	.44
Peripheral vascular disease	12 (9.0%)	14 (10.5%)	.84
STS-PROM, %	0.9 (0.5-2.1)	0.9 (0.5-1.7)	.95

Values are presented as n (%) or median [IQR] unless otherwise indicated. *BMI*, Body mass index; *STS-PROM*, Society of Thoracic Surgeons Predicted Risk of Mortality.

### Postoperative Outcomes

**Figure 3:**
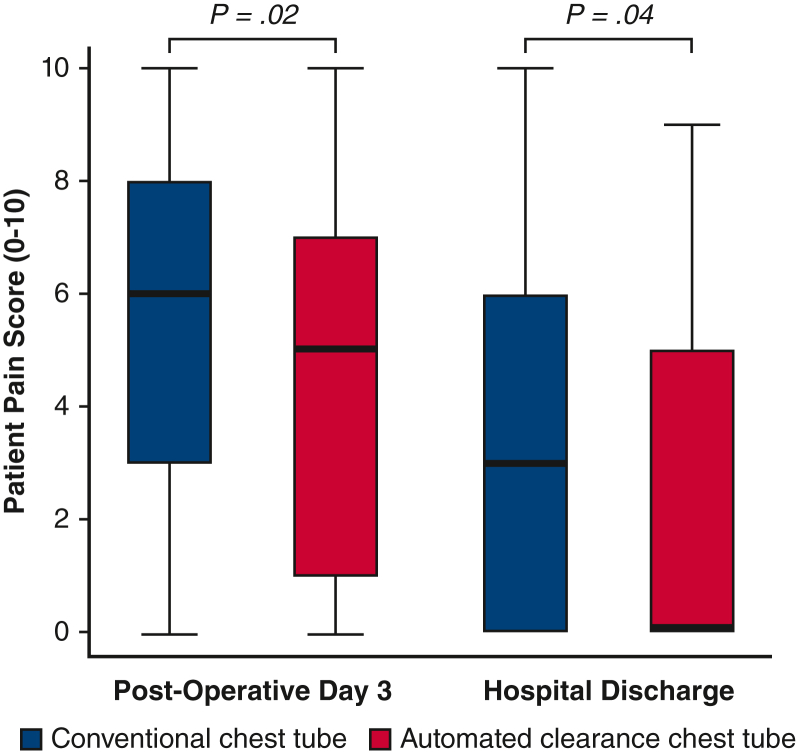
Postoperative pain profiles in patients with conventional and automated line-clearance chest tubes. Pain score results on the Integer Rating Scale (0-10) are presented in box-and-whisker plot format where the upper and lower borders of the box represent the upper and lower quartiles, the middle horizontal line represents the median, and the upper and lower horizontal lines represent the maximum and minimum values of nonoutliers.

**Figure 4:**
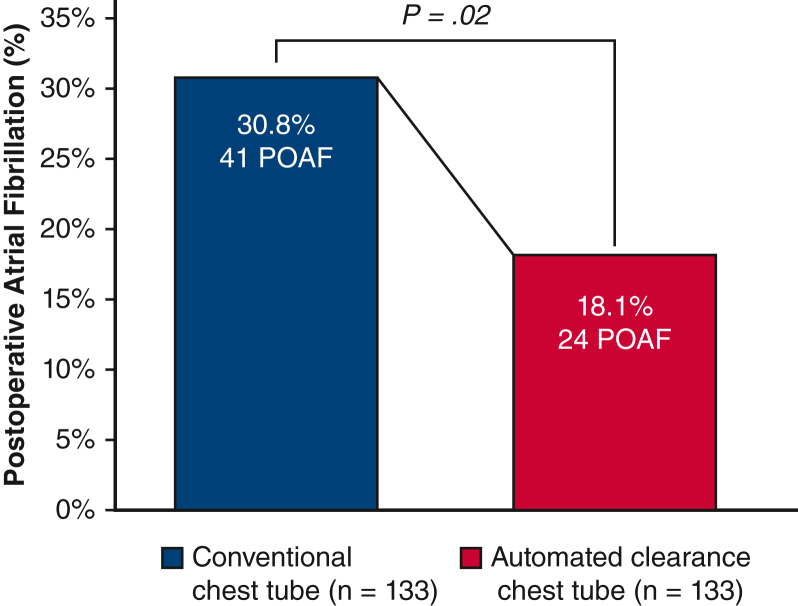
Rates of POAF in patients with conventional and automated line-clearance chest tubes. *POAF*, Postoperative atrial fibrillation.

In the matched study cohort, postoperative outcomes were notable for significant reductions in pain scores on the third postoperative day (5 [IQR, 1-7] vs 6 [IQR, 3-8]; *P* = .02) and at hospital discharge (0 [IQR, 0-5] vs 3 [IQR, 0-6]; *P* = .04) among patients receiving the 20F automated clearance chest tubes (Figure 3). Automated line-clearing chest tubes were associated with shorter duration of postoperative mechanical ventilation (5.3 hours [IQR, 4.1-6.0] vs 5.8 hours [IQR, 4.9-10.2]; *P* < .001) (Table 3). There was a significant reduction in POAF (18.1% vs 30.8%; *P* = .02) in patients receiving automated line-clearing chest tubes (Figure 4). Postoperative pleural effusions requiring drainage were present in 3 patients (2.3%) in the automated clearance chest tube group and 4 patients (3.0%) in the conventional chest tube group. No patients developed pericardial effusions requiring drainage, and no patients underwent reoperation for bleeding or tamponade. There were no significant differences in mortality, myocardial infarction, stroke, or sternal wound infections between automated line-clearing and conventional chest tubes. Postoperative outcomes in the overall unmatched study cohort were notable for higher incidence of prolonged intubation (11.3% vs 3.3%; *P* < .001), pneumonia (4.8% vs 0.5%; *P* < .01), and postoperative renal failure requiring dialysis (3.5% vs 0.5%; *P* = .03) in patients receiving conventional chest tubes versus automated line-clearance chest tubes (Table E3). Postoperative length of stay was shorter in the overall cohort of patients with automated clearance chest tubes compared with those with conventional chest tubes (6 days [IQR, 5-8] vs 7 days [IQR, 5-11; *P* < .001).

**Table 3:** Operative details and hospital outcomes (matched)

Variable	Conventional patients (n = 133)	Automated clearance (n = 133)	*P* value
Intraoperative details			
CPB time, min	103.4 ± 43.8	112.6 ± 31.3	.06
Crossclamp time, min	63.5 ± 29.2	82.6 ± 24.2	<.01
Death	3 (2.3%)	1 (0.8%)	.62
MI	0 (0%)	1 (0.8%)	>.99
Stroke	5 (3.8%)	3 (2.3%)	.72
Reoperation for bleeding	0 (0%)	0 (0%)	-
Pain score			
Baseline	0 (0-0)	0 (0-0)	-
Postoperative day 3	6 (3-8)	5 (1-7)	.02
Discharge	3 (0-6)	0 (0-5)	.04
Postoperative ventilation duration, h	5.8 (4.9-10.2)	5.3 (4.1-6.0)	<.001
Prolonged intubation >24 h	9 (6.8%)	4 (3.0%)	.26
Pneumonia	6 (4.5%)	1 (0.8%)	.12
Postoperative drainage			
Pericardial effusion	0 (0%)	0 (0%)	-
Pleural effusion	4 (3.0%)	3 (2.3%)	>.99
POAF	41 (30.8%)	24 (18.1%)	.02
Renal failure requiring dialysis	1 (0.8%)	1 (0.8%)	>.99
Deep sternal wound infection	2 (1.5%)	0 (0%)	.50
Postoperative LOS, d	6 (5-9)	6 (5-8)	.12

Values are presented as n (%) or median [IQR] unless otherwise indicated. *CPB*, Cardiopulmonary bypass; *MI*, myocardial infarction; *POAF*, postoperative atrial fibrillation; *LOS*, length of stay.

## Discussion

In the present study, we examine our institutional experience with the use of a new chest drainage technology in adult cardiac surgery and evaluate its effect on postoperative pain and recovery. The Centese Thoraguard chest drainage system incorporates both digital output monitoring and an automated active clearance mechanism to maintain chest tube patency and has been adopted for use in routine cardiac surgery at our institution.^6^ In a matched study cohort of patients with similar preoperative characteristics, the use of Thoraguard automated line-clearing chest tubes was associated with less postoperative pain and shorter duration of mechanical ventilation compared with conventional analog chest tubes. There was a reduced incidence of POAF of 18.1% in patients with automated clearance chest tubes versus 30.8% in patients with conventional chest tubes. In the overall study population, patients with automated line-clearance chest tubes had a median 1-day reduction in postoperative length of stay; however, no differences were observed after matching for preoperative characteristics.

Chest tube occlusion is a common occurrence affecting up to 36% of patients undergoing cardiac surgery and has been recognized to clinically manifest in the form of complications resulting from RBS.^1^, ^2^, ^3^, ^4^, ^5^ Although the maintenance of chest tube patency has been emphasized in Enhanced Recovery After Surgery guidelines to enhance recovery after cardiac surgery, traditional methods for clearing obstructed chest tubes, such as milking, stripping, or introducing external suction, have been questioned on their efficacy, sterility, and potential to cause tissue injury.^8^ In recent years, active clearance chest drains have emerged as potential solutions to more effectively maintain tube patency and are being investigated for their efficacy in reducing postoperative complications.^9^, ^10^, ^11^, ^12^, ^13^ The Thoraguard system used in this study differs from other commercially available active clearance chest tubes in its use of an air bolus mechanism for tube clearance as well as its automation of the active clearance procedure to sweep chest tubes every 5 minutes without bedside staff intervention.

The clinical benefits associated with automated line-clearance observed in this study were well corroborated by existing literature investigating outcomes in other active clearance chest tube systems. In particular, multiple retrospective and prospective studies have all separately demonstrated reductions in POAF and hospital length of stay using active clearance chest tubes. In our study, we demonstrated a reduced POAF rate of 18.1% with automated clearance tubes compared with 30.8% with conventional drains, aligning with prior studies reporting POAF rates of 20% to 25% with active clearance versus 30% to 38% with conventional chest tubes.^9^^,^^13^^,^^14^ Although several prior studies of active clearance chest drainage to date have also reported 1-day reductions in hospital length of stay, this difference did not persist in our study cohorts after matching for preoperative characteristics.^13^, ^14^, ^15^ Lastly, the reduction in postoperative pain observed in our study has not been specifically investigated before in the context of active clearance chest drainage. It is possible that improved pain control with Thoraguard chest tubes is related to their smaller size (20F) compared with conventional chest tubes (24F-40F), a property that is facilitated by but not directly related to their active clearance mechanism.

In addition to clinical outcomes associated with active chest tube clearance, the digital monitoring function of the Thoraguard chest tube system has been noted to be beneficial in daily chest tube management at our institution. With automated hourly measurements of chest tube output volumes, bedside nursing demands can be alleviated through simplified charting and recording. Although our present work did not include a thoracic surgery population, the digital quantification of air leaks with electronic chest tube systems has been studied in patients undergoing lung resection to promote earlier removal of chest tubes from the pleural space.^16^, ^17^, ^18^, ^19^, ^20^, ^21^, ^22^ In a recent study of chest tube management after lobectomy and segmentectomy by McCormack and colleagues,^23^ adoption of the Thoraguard chest tube system facilitated an increased rate of safe early chest tube removal within 12 hours of surgery from 25% to 30% to 78% to 93%. The dual functionality of the Thoraguard system in both digital drainage monitoring and active drain clearance presents a unique advancement in chest tube technology and invites further investigation to explore its potential benefits in a broader range of surgical populations.

### Study Limitations

The findings of the present study must be considered in the context of several limitations. First, this was a retrospective and single-center experience that did not involve patient randomization to the use of automated clearance or conventional chest tubes. Although the possibility of selection bias was minimized through matching across preoperative patient risk factors, the potential for unmeasured hidden confounders such as differences in surgical technique remains. Given the retrospective nature of the study, postoperative pain medications were not dosed identically between groups, and likely there were multifactorial contributors including chest tube duration leading to the decreased pain observed with automated clearance chest tubes. Although it is difficult to definitively attribute differences in postoperative complications to RBS without specific measurement of undrained blood, our observed postoperative outcome improvements are commensurate with existing literature on active clearance chest tubes and support the notion of RBS as a shared mechanism for increased complications. Future research examining biomarkers and inflammatory cytokines within chest tube effluents may provide valuable insights to characterize the biologic mechanisms by which RBS can impact clinical outcomes.

## Conclusions

The adoption of the Centese Thoraguard automated line-clearing chest tube system in adult cardiac surgery at our institution was associated with significant improvements in postoperative recovery. The use of automated line-clearing chest tubes resulted in less postoperative pain, shorter duration of mechanical ventilation, and reduced incidence of POAF without increased morbidity or mortality. Automated line-clearance chest tubes may provide an important modality for maintaining chest tube patency and serve as a key component of enhanced recovery after cardiac surgery.

### Webcast

You can watch a Webcast of this AATS meeting presentation by going to: https://www.aats.org/resources/automated-line-clearing-chest-7382.

**Figure:**
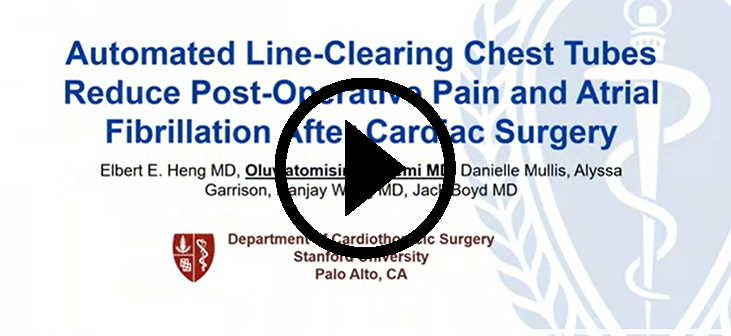

## Conflict of Interest Statement

The authors reported no conflicts of interest.

The *Journal* policy requires editors and reviewers to disclose conflicts of interest and to decline handling or reviewing manuscripts for which they may have a conflict of interest. The editors and reviewers of this article have no conflicts of interest.

## Figures and Tables

**Table E1:** Operative case breakdown (overall)

Operation	Automated clearance (n = 184)
CABG	122
Myocardial bridge unroofing	22
AVR	1
MVR	4
AVR/CABG	6
MVR/CABG	1
AVR/MVR	2
MVr	4
MVr/CABG	2
TVr	3
AVR/TVr	1
MVR/TVr	2
MVr/TVr	1
TVR/PVR	1
CABG + aorta	1
AVR + aorta	5
AVR/CABG + aorta	1
Other	5

*CABG*, Coronary artery bypass grafting; *AVR*, aortic valve replacement; *MVR*, mitral valve replacement; *MVr*, mitral valve repair; *TVr*, tricuspid valve repair; *TVR*, tricuspid valve replacement; *PVR*, pulmonary valve replacement.

**Table E2:** Baseline patient characteristics (unmatched)

Variable	Conventional patients (n = 1587)	Automated clearance (n = 184)	*P* value
Age, y	62.1 ± 13.2	63.4 ± 12.5	.20
Female	544 (34.3%)	55 (29.9%)	.25
BMI, kg/m^2^	28.0 ± 6.0	28.0 ± 6.1	.97
Hypertension	1261 (79.5%)	150 (81.5%)	.56
Heart failure	797 (50.2%)	69 (37.5%)	<.01
Ejection fraction, %	55.0 ± 11.2	56.1 ± 10.8	.22
Current smoker	157 (9.9%)	11 (6.0%)	.11
Chronic lung disease	229 (14.4%)	23 (12.5%)	.58
Diabetes	406 (25.6%)	79 (42.9%)	<.001
Dialysis	67 (4.2%)	8 (4.4%)	.84
Peripheral vascular disease	113 (7.1%)	15 (8.1%)	.55
STS-PROM, %	1.1 (0.5-2.7)	0.9 (0.5-1.8)	.40

Values are presented as n (%) or median [IQR] unless otherwise indicated. *BMI*, Body mass index; *STS-PROM*, Society of Thoracic Surgeons Predicted Risk of Mortality.

**Table E3:** Operative details and hospital outcomes (unmatched)

Variable	Conventional patients (n = 1587)	Automated clearance (n = 184)	*P* value
Intraoperative details			
CPB time, min	147.8 ± 81.4	113.5 ± 36.0	<.01
Crossclamp time, min	101.6 ± 61.1	83.6 ± 27.3	<.01
Death	49 (3.1%)	1 (0.5%)	.06
MI	12 (0.8%)	1 (0.5%)	>.99
Stroke	39 (2.5%)	5 (2.7%)	.80
Reoperation for bleeding	30 (1.9%)	0 (0%)	.07
Pain score			
Baseline	0 (0-0)	0 (0-0)	-
Postoperative day 3	6 (3-8)	6 (2-7)	.04
Discharge	3 (0-6)	2 (0-6)	.17
Postoperative ventilation duration, h	5.9 (4.2-12.5)	5.2 (3.7-5.9)	<.001
Prolonged intubation >24 h	180 (11.3%)	6 (3.3%)	<.001
Pneumonia	76 (4.8%)	1 (0.5%)	<.01
Postoperative drainage			
Pericardial effusion	7 (0.4%)	1 (0.5%)	.59
Pleural effusion	83 (5.2%)	4 (2.1%)	.07
POAF	339 (21.4%)	31 (16.9%)	.18
Renal failure requiring dialysis	55 (3.5%)	1 (0.5%)	.03
Deep sternal wound infection	8 (0.5%)	0 (0%)	>.99
Postoperative LOS, d	7 (5-11)	6 (5-8)	<.001

Values are presented as n (%) or median [IQR] unless otherwise indicated. *CPB*, Cardiopulmonary bypass; *MI*, myocardial infarction; *POAF*, postoperative atrial fibrillation; *LOS*, length of stay.
